# Supplementary material for: WordCommentsAnalyzer: A windows software tool for qualitative research
Source: F1000Res. 2018 Sep 4;7:536. Originally published 2018 May 3. [Version 2] doi: 10.12688/f1000research.14819.2 (PMC6137411; doi:10.12688/f1000research.14819.2)
Supplement: Supplementary file 3 [file f1000research-7-17614-s0002.tgz › b2a79496-4ada-47c4-9682-a99ce867836d.zip!/Eight Iranian Health Journals/Journal of Research in Rehabilitation Sciences _33.docx]

© Journal of Research in Rehabilitation Sciences . This work is licensed under CC BY-NC 4.0 (https://creativecommons.org/licenses/by-nc/4.0/).

Volume & Issue: دوره 8، شماره 8: 1391 ویژه نامه اختلالات حرکتی

Title: بررسی تعادل ایستایی افراد مبتلا به صافی کف پا

http://jrrs.mui.ac.ir/index.php/jrrs/article/view/605

مقدمه: صافی کف پا یکی از شایع‌ترین اختلالات اسکلتی است که علاوه بر عملکرد پا، بر تنظیم راستای مفاصل مچ، زانو و هیپ نیز تأثیر می‌گذارد. بر اساس مطالعات موجود، الگوی راه رفتن افراد مبتلا به صافی کف پا با افراد سالم متفاوت است، اما تفاوت‌های موجود در تعادل و تأثیر کفی طبی بررسی نشده است. بنابراین هدف از این مطالعه، بررسی تعادل افراد مبتلا به صافی کف پا، با و بدون استفاده از کفی طبی بود. مواد و روش‌ها: در مطالعه کارآزمایی بالینی حاضر، تعداد 40 نفر از دانشجویان دختر دانشگاه علوم پزشکی اصفهان در دو گروه سالم و مبتلا به صافی کف پا شرکت داشتند. برای اندازه‌گیری مرکز فشار از یک صفحه نیروی Kistler استفاده شد. تعادل با محاسبه نوسان مرکز فشار، طول مسیر و سرعت در راستای داخلی- خارجی و قدامی- خلفی به دست آمد. تفاوت بین دو گروه با و بدون کفی طبی چرمی توسط آزمون Two sample t و Paired t ارزیابی شد (05/0 = α). یافته‌ها: تفاوت قابل توجهی در تعادل بین افراد سالم و افراد مبتلا به صافی کف پا بر اساس سرعت مرکز فشار در صفحات قدامی- خلفی و داخلی- خارجی و سرعت کل مشاهده شد. مقدار میانگین سرعت کل مرکز فشار طی ایستادن با و بدون کفی طبی، 160 ± 42/1645 و 683 ± 2752 میلی‌متر بر دقیقه بود (05/0 > P). نتیجه‌گیری: افراد مبتلا به صافی کف پا به دلیل از دست دادن تنطیم راستای ساختار پا و کاهش درک حرکتی لیگامان‌ها و تاندون‌های پا در مقایسه با افراد سالم، تعادل کمتری طی ایستادن دارند. به نظر می‌رسد که کفی طبی سبب بهبود تنظیم راستای ساختار پا و کاهش نیروهای وارد بر لیگامان‌ها و در نتیجه بهبود تعادل می‌شود.

Title: تأثیر کوتاه مدت ویبراسیون کل بدن بر منحنی فراخوانی رفلکس H عضله سولئوس

http://jrrs.mui.ac.ir/index.php/jrrs/article/view/872

مقدمه: بر خلاف اثرات تسهیلی بیان شده پس از اعمال ویبراسیون کل بدن (Whole body vibration یا WBV)، نتایج برخی مطالعات افزایش مهار پاسخ‌های عضلانی را نشان می‌دهد. هدف مطالعه حاضر، بررسی پاسخ کوتاه مدت منحنی فراخوانی رفلکس H عضله سولئوس پس از اعمال یک جلسه WBV بود. مواد و روش‌ها: 26 زن سالم غیر ورزشکار (سن 85/2 ± 11/26 سال) داوطلبانه در مطالعه شرکت کردند. افراد در دو جلسه آزمون و شم با حفظ وضعیت نیمه اسکوات بر روی صفحه ویبراسیون قرار گرفتند. در جلسه آزمون ویبراسیون با فرکانس 30 هرتز و دامنه 3 میلی‌متر به صورت 3 دوره 1 دقیقه‌ای اعمال شد. قبل، بلافاصله، 5 و 10 دقیقه بعد از اعمال ویبراسیون منحنی فراخوانی رفلکس H از عضله سولئوس ثبت شد. یافته‌ها: در جلسه شم و آزمون شدت تحریک آستانه منحنی و شدت لازم برای ثبت دامنه حداکثر بلافاصله بعد از WBV افزایش معنی‌دار نشان داد (05/0 > P). دامنه رفلکس H ابتدایی در هر دو جلسه و دامنه Hmax در جلسه شم کاهش معنی‌داری نشان داد (05/0 > P). آنالیز پارامترهای مربوط به آستانه و قله منحنی فراخوانی رفلکس H بین دو جلسه آزمون و شم تفاوت معنی‌داری نشان نداد (05/0 < P). نتیجه‌گیری: با توجه به جابه‌جایی منحنی فراخوانی به سمت راست و پاسخ مهاری در دو جلسه شم و آزمون، به نظر می‌رسد حفظ وضعیت نیمه اسکوات و مکانیسم‌های کنترلی فعال شده مانند انقباض عضلات اکستانسوری در مهار منحنی فراخوانی رفلکس H مؤثر باشد و اعمال ویبراسیون نیز هیچ اثر تسهیلی بر این پاسخ مهاری نداشته است.

Title: ارزیابی اثربخشی گروه درمانی آموزشی- روانی بر ارتقای سلامت عمومی خانواده‌های بیماران مبتلا به اختلالات دو قطبی: یک مطالعه پایلوت

http://jrrs.mui.ac.ir/index.php/jrrs/article/view/721

مقدمه: مراقبین خانوادگی بیماران روان در واقع اساس سیستم مراقبت‌های بهداشتی- درمانی می‌باشند و مراقبت از بیمار مبتلا به اختلال روان‌پزشکی مسؤولیتی است که فشار و استرس زیادی برای اعضای خانواده به همراه دارد. پژوهش حاضر با هدف، بررسی اثربخشی گروه درمانی آموزشی- روانی بر ارتقای سلامت عمومی خانواده بیماران مبتلا به اختلالات دو قطبی انجام شد. مواد و روش‌ها: این پژوهش یک طرح شبه آزمایشی با گروه شاهد و انتخاب تصادفی بود. در این پژوهش، 6 نفر به عنوان گروه آزمایش و 9 نفر به عنوان گروه شاهد شرکت داشتند. کلیه شرکت کنندگان که اعضای خانواده بیماران مبتلا به اختلالات دو قطبی را تشکیل می‌دادند، پرسش‌نامه سلامت عمومی (General Health Questionnaire یا 28-GHQ) را تکمیل نمودند. گروه آزمایش به مدت 12 جلسه در جلسات گروه درمانی آموزشی- روانی شرکت داشتند، ولی گروه شاهد در این مدت هیچ گونه مداخله‌ای دریافت نکردند. همچنین به منظور تحلیل داده‌ها از آزمون ANCOVA استفاده شد. یافته‌ها: افزایش معنی‌داری در میانگین نمره سلامت عمومی گروه آزمایش مشاهده گردید (001/0 > P). نتیجه‌گیری: گروه درمانی آموزشی- روانی می‌تواند سطح سلامت عمومی خانواده‌های بیماران مبتلا به اختلال دو قطبی را بهبود بخشد.

Title: بررسی تأثیر باز و بسته بودن چشم بر پتانسیل عضلانی برانگیخته دهلیزی

http://jrrs.mui.ac.ir/index.php/jrrs/article/view/354

مقدمه: پتانسیل عضلانی برانگیخته دهلیزی، آزمون قابل اطمینان و جدیدی است که با فراهم آوردن اطلاعات تشخیصی در مورد عملکرد ساکول و یا عصب دهلیزی تحتانی، مجموعه آزمون‌های تعادلی حاضر را تکمیل نموده و سلامت رفلکس ساکولوکولیک را ارزیابی می‌نماید. دامنه پتانسیل عضلانی برانگیخته دهلیزی متناسب با سطح فعالیت الکترومیوگرافی زمینه می‌باشد که این امر لزوم حفظ ثبات فشار عضله در حین ثبت را نشان می‌دهد. تغییر در وضعیت چشم باعث تغییر در فعالیت عضله جناغی- چنبری- پستانی و بنابراین تغییر در دامنه پتانسیل عضلانی برانگیخته دهلیزی می‌گردد. از این رو، پژوهش حاضر با هدف، بررسی امکان تأثیر باز و بسته بودن چشم بر پارامترهای پتانسیل عضلانی برانگیخته دهلیزی صورت گرفت. مواد و روش‌ها: در مطالعه مقطعی- مقایسه‌ای حاضر، پتانسیل عضلانی برانگیخته دهلیزی با ارایه محرک Tone-Burst 500 هرتز با شدت dBnHL 95 در 40 فرد هنجار در محدوده سنی 30-18 سال ثبت شد. یافته‌ها: میانگین دامنه و زمان نهفتگي امواج 13P و 23N در هر دو گوش، اختلاف آماری معنی‌داری را نشان نداد (05/0 < P). نتیجه‌گیری: باز و بسته بودن چشم‌ها تأثیری بر پتانسیل عضلانی برانگیخته دهلیزی نداشت، بنابراین در صورت لزوم و جهت حذف آرتیفکت (Artifact) چشمی می‌توان آزمون پتانسیل عضلانی برانگیخته دهلیزی را با چشم بسته ثبت نمود.

Title: تأثیر تلفیق محدودیت درمانی با آموزش فشرده دو دستی بر کارکرد اندام فوقانی کودکان با فلج مغزی نیمه بدن 10-5 سال

http://jrrs.mui.ac.ir/index.php/jrrs/article/view/546

مقدمه: محدودیت درمانی و درمان فشرده دو دستی، روش‌های درمانی نویدبخشی در بهبود کارکرد اندام فوقانی کودکان با فلج مغزی نیمه بدن می‌باشند. محدودیت درمانی شامل: محدودیت اندام سالم و انجام تمرینات فشرده با اندام مبتلا می‌باشد و درمان فشرده دو دستی، مکملی برای درمان‌های دیگر اندام فوقانی می‌باشد و هدف آن بهبود هماهنگی دو دست با استفاده از تمرینات ساختار یافته است که درون فعالیت‌های کارکردی و بازی‌های دو دستی گنجانده شده است. هدف از این مطالعه، بررسی تأثیر تلفیق محدودیت درمانی با درمان فشرده دو دستی بر کارکرد اندام فوقانی کودکان با فلج مغزی نیمه بدن بود. مواد و روش‌ها: در کارآزمایی بالینی حاضر، 25 کودک 10-5 ساله به روش تصادفی ساده به دو گروه درمان و شاهد تقسیم شدند. گروه شاهد خدمات رایج در کاردرمانی را دریافت نمودند و گروه درمان علاوه بر خدمات رایج در کاردرمانی، تلفیق محدودیت درمانی و درمان فشرده دو دستی را به صورت دریافت نمودند که ابتدا اندام فوقانی سالم با محدود کننده (اسلینگ) به مدت 3 ساعت بسته شد و کودکان تکالیف داده شده را تنها با اندام مبتلا انجام دادند، سپس اسلینگ را درآورده و کودکان تکالیف داده شده را به مدت 3 ساعت با هر دو دست تمرین نمودند .این روند به مدت 10 روز در طی دو هفته متوالی ادامه یافت. کارکرد اندام فوقانی و تون عضلانی کودکان با استفاده از آزمون کارکرد حرکتی جبسن- تیلور (Jebsen-Taylor test of hand function) و مقیاس اصلاح شده آشورث (Modified Ashwort scale) پیش و پس از مداخله درمانی مورد سنجش قرار گرفت. یافته‌ها: کارکرد حرکتی کودکانی که تحت درمان تلفیق محدودیت درمانی با درمان فشرده دو دستی قرار گرفتندف نسبت به گروه شاهد تفاوت معنی‌داری داشت (05/0 > P)، اما میزان اسپاستیسیته عضلانی بعد از انجام مداخله درمانی تفاوت معنی‌داری نداشت (05/0 < P). نتیجه‌گیری: تلفیق محدودیت درمانی با درمان فشرده دو دستی موجب بهبود کارکرد حرکتی کودکان با فلج مغزی نیمه بدن می‌شود..

Title: بررسی نگرش متخصصین و کارشناسان کادر توان‌بخشی شهر شیراز نسبت به فرایند کار تیمی

http://jrrs.mui.ac.ir/index.php/jrrs/article/view/552

مقدمه: توان‌بخشی فرایندی مستمر با طیفی از تخصص‌های مختلف است. یکی از جنبه‌های مهم این فرایند، کار تیمی است. تیم توان‌بخشی شامل متخصصین طب فیزیکی، فیزیوتراپیست، کاردرمانگر، گفتار درمانگر و کارشناس اعضای مصنوعی است، بنابراین نگرش این اعضا نسبت به نحوه انجام کار تیمی تأثیر بسزایی در کیفیت فرایند توان‌بخشی خواهد داشت. هدف این مطالعه، بررسی نگرش متخصصین یاد شده نسبت به کار تیمی بود. مواد و روش‌ها: مطالعه حاضر از نوع توصیفی- مقطعی بود. روش نمونه‌گیری به شیوه سرشماری و جامعه آماری شامل دستیاران و متخصصین طب فیزیکی، کارشناسان کاردرمانی، گفتار درمانی، فیزیوتراپی و اعضای مصنوعی شاغل در مراکز درمانی خصوصی و دولتی شهر شیراز بود. ابزار جمع‌آوری اطلاعات پرسش‌نامه محقق ساخته نگرش تیمی بود. ضریب Cronbach¢s alpha برای بررسی پایایی پرسش‌نامه، تحلیل عاملی برای بررسی ویژگی‌های ساختاری پرسش‌نامه و آزمون t برای بررسی وضعیت نگرش مورد استفاده قرار گرفت. یافته‌ها: ضریب Cronbach¢s alpha (87/0) مؤید پایایی خوب پرسش‌نامه بود. بر اساس یافته‌های مطالعه تمامی گروه‌های شغلی از نگرش مثبتی نسبت به کار تیمی برخوردار بودند (05/0 > P). با توجه به برآورد درصد پاسخ داده شده به سؤالات پرسش‌نامه، عوامل مؤثر و مخاطره‌آمیز در روند کار تیمی از دیدگاه افراد استخراج گردید. نتیجه‌گیری: رویکرد بیمارمحور، آگاهی کافی نسبت به دانش و مهارت سایر اعضا و رعایت ارزش‌های اخلاقی و حرفه‌ای برخی عوامل مؤثر در بهبود کار تیمی و اختلافات شخصی اعضا، ارتباطات درون گروهی ضعیف، پنداشت نامناسب از درخواست کمک و عدم آگاهی کافی نسبت به روند انجام کار گروهی از عوامل مخاطره‌آمیز می‌باشد.

Title: اثر صافی کف پا بر الگوی فعالیت عضلانی اندام تحتانی و ویژگی‌های فشار کف پایی هنگام راه رفتن

http://jrrs.mui.ac.ir/index.php/jrrs/article/view/537

مقدمه: اثر وضعیت غیر طبیعی پا بر عملکرد حرکتی اندام تحتانی به ویژه در بین کودکان و نوجوانان کمتر مورد توجه قرار گرفته است. هدف مطالعه حاضر، مقایسه الگوی فعالیت عضلات منتخب اندام تحتانی و ویژگی‌های فشار کف پایی پسران نوجوان دچار صافی کف پا منعطف با گروه نرمال هنگام راه رفتن بود. مواد و روش‌ها: تعداد ۱۲ نفر نوجوان با ساختار صافی کف پا و ۱۲ نفر با ساختار پای نرمال با استفاده از روش شاخص قوس طولی میانی پا در این مطالعه شرکت کردند.  فعالیت الکترومایوگرافی عضلات تیبیالیس انتریور، پرونئوس لانگوس، سولئوس، بخش خارجی و داخلی عضله گاستروکنیمیوس به همراه ویژگی‌های فشار کف پایی آزمودنی‌ها هنگام راه رفتن با پای برهنه در یک مسیر ۱۰ متری با سرعت خودانتخابی جمع‌آوری شد. داده‌ها با روش آماری Independent t تجزیه و تحلیل گردید. یافته‌ها: فعالیت الکتریکی قسمت داخلی و خارجی عضله دو قلو طی مرحله انتقال وزن در افراد با پای نرمال نسبت به گروه با صافی کف پا به طور معنی‌داری بیشتر بود (001/0 = P)؛ در حالی که قسمت داخلی گاستروکنیمیوس در مرحله انتهای استانس فعالیت کمتری (048/0 = P) داشت. حداکثر فشار وارد شده در نواحی انگشت شست، انگشتان دوم تا پنجم، متاتارسال دوم و سوم و قسمت داخلی پاشنه در افراد دچار صافی کف پا بیشتر بود. به علاوه میزان تغییرات مرکز فشار پا در مراحل انتقال وزن، مید استانس و کل مرحله استانس، بین دو گروه تفاوت مشاهده شد (به ترتیب: 011/0 = P، 015/0 = P و 050/0 = P). نتیجه‌گیری: وضعیت غیر طبیعی پا می‌تواند بر تغییر فعالیت عضلات اندام تحتاني و ویژگی‌های توزیع فشار کف پایی هنگام راه رفتن مؤثر باشد. نتایج این تحقیق بر لزوم بررسی دقیق و جامع وضعیت غیر طبیعی پا بر عملکرد اندام تحتانی هنگام راه رفتن برای طراحی برنامه درمانی مناسب تأکید داشت.

Title: ارزیابی مزایا و معایب کراچ و واکر: مروری بر مقالات

http://jrrs.mui.ac.ir/index.php/jrrs/article/view/941

مقدمه: تعداد افرادی که از وسایل کمکی برای ایستادن و راه رفتن استفاده می‌کنند در حال افزایش است. انواع مختلفی از واکر و کراچ برای بهبود عملکرد افراد طراحی شده است، ولی تجویز این وسایل بر طبق ملاک‌هایی است که به لحاظ کلینیکی آزمایش نشده است. به علاوه تفاوت بین کراچ و واکر واضح نیست، این‌که کدام نوع از واکر مناسب است و همچنین تفاوت بین طراحی‌های جدید و قدیمی واکر و کراچ موضوعی است که به آن پرداخته نشده است. بنابراین هدف از مقاله مروری حاضر پاسخ به سؤالات ذکر شده بود. مواد و روش‌ها: یک جستجوی الکترونیک در پایگاه‌های اطلاعاتی PubMed، ISI web of science و Embase از سال 1960 تا 2012 انجام گردید. عنوان و خلاصه مقالات بر طبق معیارهای انتخاب ارزیابی شدند. کیفیت مقالات از طریق ابزار Black and Down مورد ارزیابی قرار گرفت. یافته‌ها: حدود 24 مقاله برای تحلیل نهایی انتخاب شدند. کیفیت اکثر مطالعات پایین بود و بیشتر تحقیقات روی نمونه‌های طبیعی انجام شده بود. به علاوه، فقط بعضی پارامترها مثل نیروی اعمالی به پا در حین راه رفتن و مصرف انرژی ارزیابی گردید است. شواهد در دسترس برای تشخیص عملکرد واکر و کراچ کافی نیست. نتیجه‌گیری: بر طبق مطالعات تحقیقی انجام شده، تفاوت بین انواع مختلف واکر و کراچ و مزایای واکر نسبت به کراچ واضح نیست. تأثیر طول کراچ و واکر بر کارایی نمونه‌ها بحث‌برانگیز است. به علاوه تعداد نمونه‌های اندک مطالعات، نتیجه‌گیری محکم را تحت تأثیر قرار داده است. بنابراین انجام مطالعات بزرگ‌تر برای تست و ارزیابی انواع مختلف کراچ و واکر روی افراد معلول توصیه می‌شود.

Title: پروتکل صندلی چرخان و کاربرد آن در بیماری‌های مختلف

http://jrrs.mui.ac.ir/index.php/jrrs/article/view/818

مقدمه: آزمون صندلی چرخان به منظور بسط ارزیابی‌های درگیری سیستم محیطی و بررسی روند جبران مرکزی و پیگیری تاریخچه سیر بیماری و پايش کارایی درمان‌های خاص، كاربرد دارد. پارامترهای مورد ارزیابی و آنالیز در آزمایش صندلی چرخان فاز، بهره و تقارن است. هدف از پژوهش حاضر، بررسی نتایج پژوهش‌های انجام شده در حوزه صندلی چرخان و بررسی کاربردهای بالینی آن بود. مواد و روش‌ها: مقاله مروری حاضر مقالات منتشر شده از سال 2003 تا 2009 بود که در پایگاه‌های اطلاعاتی Google Scholar, Science Direct, EBSCO, PubMed, Thieme و با استفاده از کلید واژه‌‌های صندلی چرخان، جبران مرکزی، نیستاگموس وجود داشت. یافته‌ها: حاصل جستجوی انجام گرفته 58 مقاله پژوهشی و 4 مقاله مروری بود. پس از بررسی مقالات، 20 مقاله به موضوع مورد نظر پرداخته بود و در این مطالعه مروری مورد بررسی قرار گرفت. نتیجه‌گیری: آزمون صندلی چرخان به علت مزایایی مانند فیزیولوژیک بودن محرک به کار رفته و پایش جبران توسط تغییرات تقارن و نیز قابل اجرا بودن آزمون، زمانی که بیمار حتی از داروهای مهارکننده وستیبولی استفاده می‌کند و راحتی آزمون، امروزه می‌تواند در پروتکل ارزیابی‌های دهلیزی قرار گیرد.

Title: مروری بر شاخص‌های کینتیکی در افراد مبتلا به اسکولیوز

http://jrrs.mui.ac.ir/index.php/jrrs/article/view/950

مقدمه: اسکولیوز به عنوان یک بدشکلی رایج ستون فقرات محسوب می‌شود. بسیاری از مطالعات پیشین، آنالیز حرکات افراد مبتلا به اسکولیوز را بر پایه اندازه‌گیری‌های کینماتیک بررسی کردند، اما از آن‌جایی که اسکولیوز یک بدشکلی ساختاری است که تقارن ستون فقرات را به هم می‌زند و در نتیجه باعث تغییر مکان مرکز جرم (center of mass یا COM) و توزیع نامتقارن وزن در اندام تحتانی می‌شود. بررسی و ارزیابی اطلاعات کینتیکی باعث فهم بهتر علل اسکولیوز می‌شود. هدف این تحقیق، بررسی شاخص‌های کینتیکی در گیت افراد مبتلا به اسکولیوز و تفاوت آن با افراد سالم با توجه به مطالعات گذشته است. مواد و روش‌ها: در روند این تحقیق از سایت‌های Google scholar، PubMed، Elsevier، Ovid و از کلمات کلیدی Scoliosis، Kinetic، نیروی عکس‌العمل زمین (Ground reaction force)، مرکز فشار (Center of pressure)، گشتاور (Moment) و نیروهای بین مهره‌ای (Intervertebral force) و در این بین مقالات از سال 1984 تا 2012 انتخاب و استفاده گردید. در این تحقیق کیفیت مقالات نیز بر حسب جدول Black and down tools بررسی و امتیازگذاری شدند. یافته‌ها: با استفاده از روش جستجوی ذکر شده، 13 مقاله به دست آمد که با توجه به معیارهای ورود و خروج، از 11 مقاله استفاده شد. در بین بررسی‌های انجام شده بر روی شاخص‌های تقارن، محققان یک عدم تقارن را مشاهده کردند، اما این عدم تقارن الگوی خاصی را متناسب با انحنا دنبال نمی‌کرد. در بررسی که بر جابجایی مرکز فشار انجام شد به این نتیجه رسیدند که جابجایی بیشتر در جهت داخل- خارج است. مطالعه دیگری ارزیابی اطلاعات کینتیکی را حین گام برداشتن به پهلو پیشنهاد کرد؛ چرا که در این حالت تفاوت در عدم تقارن شاخص‌ها بین دو گروه شاهد و اسکولیوزی بیشتر بود. نتیجه‌گیری: در آخر با بررسی مطالعات گذشته، یک عدم تقارن در شاخص‌ها دیده شد، اما هنوز رابطه بین این شاخص‌ها و میزان انحنا در این افراد یافت نشده است.

Title: تاثیر ورزش در درمان اسکولیوز با علت ناشناخته: یک مطالعه مروری

http://jrrs.mui.ac.ir/index.php/jrrs/article/view/942

مقدمه: اسکولیوز با علت ناشناخته یک ناهنجاری سه بعدی در ستون فقرات است که بیشتر در دختران نوجوان روی می‌دهد. اگر که اسکولیوز بدون درمان رها شود، می‌تواند پیشرفت کند و منجر به ایجاد درد کمر، نقص در ظرفیت حیاتی و اختلالات روانی- اجتماعی گردد. این چنین به نظر می‌رسد که درمان‌های ورزش بنیان می‌توانند در درمان اسکولیوز مؤثر واقع شوند، بنابراین هدف اولیه در مطالعه مروری حاضر، ارزیابی تأثیرگذاری ورزش‌های خاص اسکولیوز در درمان اسکولیوز با علت ناشناخته (Idiopathic) بود. مواد و روش‌ها: جستجوی انجام شده در پایگاه‌های اطلاعاتی بین‌المللی Ebsco (Medline از طریق PubMed)، ISI Web of Knowledge  صورت گرفت. در این جستجو از اصطلاحاتی مانند: اسکولیوز همراه با مرور، اسکولیوز همراه با تمرین ورزشی (Exercise)، اسکولیوز همراه با ورزش (Sport) و اسکولیوز همراه با توان‌بخشی استفاده گردید. معیارهای ورود به مطالعه حاضر شامل موارد زیر می‌باشد: 1) بیماران فقط با فرایند استفاده از ورزش‌های فیزیکی درمان شده باشند. 2) طراحی مطالعه در قالب بازبینی سیستماتیک باشد. یافته‌ها: سرانجام پنج مقاله بر طبق معیارهای ورود بررسی گردید. از پنج مقاله باقی‌مانده سه مورد به دلیل کمبود کارآزمایی تصادفی شاهددار با کیفیت بالا در این زمینه، استفاده از ورزش را در درمان اسکولیوز با علت ناشناخته مورد حمایت قرار نداد، ولی دو بازبینی سیستماتیک در سال‌های 2008 و 2011  مؤثر بودن ورزش را در کاهش نرخ پیشرفت و بهبود زاویه انحراف به اثبات رسانید. نتیجه‌گیری: نتایج نویدبخش حاصل از مطالعات ذکر شده یک بنیان را برای این فرضیه که «ورزش فیزیکی در درمان اسکولیوز با علت ناشناخته تأثیرگذار است» فراهم می‌آورند. فرضیه‌ای که هر چند باید به وسیله یک کارآزمایی تصادفی شاهددار با طراحی مناسب، مورد بازبینی قرار گیرد.

Title: بررسی الگوی راه رفتن و تعادل بیماران مبتلا به اسکولیوز بر اساس بررسی متون

http://jrrs.mui.ac.ir/index.php/jrrs/article/view/945

مقدمه: اسکولیوز (Scoliosis)، یک ناهنجاری سه بعدی ستون فقرات و قفسه سینه است که منجر به تغییر ارتباط بین اجزای سیستم اسکلتی بدن، آناتومی ستون فقرات و تقارن تنه شده و باعث تغییر الگوی راه رفتن بیماران می‌شود. اسکولیوز موجب تغییر وضعیت مرکز ثقل بدن و نحوه‌ توزیع وزن بر اندام تحتانی می‌گردد. بنابراین می‌توان استنباط کرد که بیماران مبتلا به اسکولیوز تعادل کم‌تری نسبت به همسالان سالم خود دارند مواد و روش‌ها: جستجوی مقالات مرتبط با اسکولیوز و تأثیر این ناهنجاری بر پارامترهای کینتیکی و کینماتیکی و نیز تأثیر بریس بر پارامترهای ذکر شده با استفاده از سایت‌های جستجوگر PubMed، Google scholar و ISI web of knowledge انجام گرفت. مقالات مرتبط با این حوزه بین سال‌های 1998 تا 2012 منتشر شده بودند. یافته‌ها: با استفاده از کلمات کلیدی مدنظر 54 مقاله به دست آمد. 26 مقاله بر اساس عنوان و چکیده،‌ مطابق با موضوع تحقیق بود و 6 مقاله‌ دیگر با بررسی رفرنس مقالات انتخاب شده در مطالعه شرکت داده شدند. مقالات محدودی تأثیر بریس را بر متغیرهای کینتیکی و کینماتیکی مورد بررسی قرار بودند. نتيجه‌گيري: با بررسی مطالعات موجود نمی‌توان به نظر واحدی در مورد تأثیر اسکولیوز بر پارامترهای کینتیکی و کینماتیکی و نیز تأثیر بریس بر این پارامترها رسید.

Title: مروری بر متغیرهای کینماتیکی در افراد مبتلا به اسکولیوز

http://jrrs.mui.ac.ir/index.php/jrrs/article/view/951

مقدمه: اسکولیوز یکی از رایج‌ترین مشکلات ارتوپدی در کودکان و بزرگسالان است که علت آن مشخص نیست. اسکولیوز یک بدشکلی در سه صفحه است که در صفحه فرونتال و عرضی قابل ملاحظه می‌باشد. این بیماری با تغییراتی که در آناتومی ستون فقرات و لگن ایجاد می‌کند باعث تغییر در پارامترهای گیت می‌شود. هدف از انجام این مطالعه، بررسی تغییرات پارامترهای کینماتیکی در اندام تحتانی، ستون فقرات و اندام فوقانی افراد اسکولیوزی در مقایسه با افراد نرمال است که در اثر اسکولیوز ایجاد شده است. با مشخص شدن الگوی دقیق این تغییرات و تأثیر شدت اسکولیوز بر روی این پارامترها می‌توان از این داده‌ها برای بررسی پیشرفت و یا بهبود اسکولیوز استفاده کرد. مواد و روش‌ها: با استفاده از کلید واژه‌های اسکولیوز (Scoliosis)، گیت (Gait)، کینماتیک (Kinematic) در سایت‌های Google scholar، PubMed، Elsevier، Ovid و Springer مقالات مرتبط مشخص شد و با توجه به معیارهای ورود و خروج مقالات بررسی شدند. با استفاده از روش Black and tool down مقالات امتیازدهی شدند. یافته‌ها: با توجه به روش جستجو، 25 مقاله پیدا شد و با در نظر گرفتن معیارهای ورود و خروج 9 مقاله مورد بررسی قرار گرفت. مقالات از سال 1998 تا 2012 هستند و به صورت طرح‌های پژوهشی به مقایسه متغیرها بین افراد سالم و مبتلا اسکولیوز پرداخته‌اند. متغیرهای کینماتیکی اندام تحتانی کاهش دامنه حرکتی را نشان دادند که البته الگوی حرکتی تغییر زیادی نداشته است. بیشترین عدم تقارن در داده‌ها در تنه مشاهده شد و مشخص گردید فقط در مواردی که فرد یک انحنای بزرگ ناحیه پشتی دارد، چرخش لگن در جهت انحنا است و در موارد دیگر انحنا، تغییرات لگن قابل پیش‌بینی نیست. لگن تغییراتی نیز در عکس‌های رادیولوژی نشان می‌دهد که با توجه به ارتباط آناتومیکی بین لگن و ستون فقرات کمری انحنای ناحیه توراکولومبار و لومبار بیشترین تأثیر را در لگن دارد. نتیجه‌گیری: بیشتر مقالات به بررسی پارامترها در حرکت با سرعت‌های مختلف پرداخته‌اند. برای دستیابی به یک نتیجه جامع باید یک استاندارد از نظر نوع انحنا و شرایط گرفتن تست وجود داشته باشد، به خصوص در موارد اندام فوقانی که مطالعات کمی نیز صورت گرفته است.

Title: بررسی عملکرد یک فرد مبتلا به اسکولیوز در راه رفتن با و بدون ارتز

http://jrrs.mui.ac.ir/index.php/jrrs/article/view/921

مقدمه: اسکولیوز (Scoliosis)، یکی از ناهنجاری‌های سیستم اسکلتی- عضلانی است که بر عملکرد فرد طی راه رفتن و ایستادن اثرگذار است. اطلاعات کمی در مورد تأثیر ارتز بر پارامترهای تعادل و راه رفتن بیماران اسکولیوزی وجود دارد، بنابراین هدف از مطالعه‌ حاضر، بررسی پارامترهای ذکر شده بود. مواد و روش‌ها: در مطالعه حاضر یک دختر 8 ساله‌ مبتلا به اسکولیوز شرکت داده شد. این بیمار دارای انحنای اسکولیوزی با شدت 29 درجه و رأس انحنا در سطح 8T قرار گرفته بود. پارامترهای راه رفتن و تعادل بیمار با و بدون استفاده از ارتز با استفاده از سیستم آنالیز گیت مجهز به 7 دوربین و صفحه‌ فشار (Kistler force plate) بررسی شد. ضمن آن‌که طول تاندون عضلات ارکتور اسپاین و عضلات مایل شکمی داخلی و خارجی هنگام راه رفتن توسط نرم‌افزار Open- SIMM مورد ارزیابی قرار گرفت. یافته‌ها: به نظر می‌رسد ارتز باعث بهبود عملکرد بیماران حین راه رفتن و ایستادن می‌شود، ضمن آن‌که باعث کشش عضلات پارااسپینال در سمت تقعر انحنای اسکولیوزی می‌شود. نتیجه‌گیری: ارتز باعث تنظیم راستای ستون فقرات و بهبود عملکرد بیمار در راه رفتن و ایستادن می‌شود. از آن‌جا که ارتز باعث افزایش طول عضلات پارااسپینال می‌شود، بنابراین می‌توان استنباط کرد که ارتز می‌تواند باعث اصلاح انحنای اسکولیوزی شود.
